# Lack of MHC class I surface expression on neoplastic cells and poor activation of the secretory pathway of cytotoxic cells in oral squamous cell carcinomas

**DOI:** 10.1038/sj.bjc.6690780

**Published:** 1999-11

**Authors:** I Cruz, C J L M Meijer, J M M Walboomers, P J F Snijders, I Van der Waal

**Affiliations:** Unit of Molecular Pathology, Department of Pathology, Department of Oral & Maxillofacial Surgery/Oral Pathology/ACTA, University Hospital Vrije Universiteit, De Boelelaan 1117, 1081 HV Amsterdam, The Netherlands

**Keywords:** oral carcinoma, MHC class I, β2-microglobulin, CTLs, NK cells, granzyme B

## Abstract

Cytotoxic T lymphocytes (CTLs) and natural killer (NK) cells use the secretory pathway of perforin/granzymes to kill their target cells. In contrast to NK cells, CTL responses are MHC class I restricted. In this study we analysed the relative activation of CTL and NK cells in relation with MHC class I expression on oral squamous cell carcinomas (OSCCs). MHC class I expression was investigated in 47 OSCCs by immunohistochemistry using HCA2, HC10 and β2-m antibodies. The presence of CTLs, NK cells, and its activation, was investigated in 21 of these OSCCs using respectively, CD8, CD57 and GrB7 antibodies. The Q-Prodit measuring system was used for quantification of cytotoxic cells. All OSCCs showed weak or absent staining of β2-m on the cell surface. The absence of β2-m was significantly associated with absent expression of MHC class I heavy chain as detected by HC10 antibody (*P* = 0.004). In tumour infiltrates CTLs always outnumbered NK cells, as reflected by the ratio CD57/CD8 being always inferior to one (mean: 0.19; SD: 0.15). The proportion of activated cytotoxic cells as detected by granzyme B expression was generally low (mean: 8.6%; SD 8.9). A clear correlation between MHC class I expression and the relative proportion of NK cells/CTLs was not found. This study shows that the majority of OSCCs show weak or absent expression of MHC class I molecules on the cell surface, possibly due to alterations in the normal β2-m pathway. The low proportion of granzyme B-positive CTLs/NK cells indicates that the secretory pathway of cytotoxicity is poor in these patients. The lack of correlation between MHC class I expression and CTL/NK cell activation as detected by granzyme B expression suggests that, next to poor antigen presentation, also local factors seem to determine the final outcome of the cytotoxic immune response. © 1999 Cancer Research Campaign


British Journal of Cancer (1999) 81(5), 881–889
© 1999 Cancer Research Campaign
Article no. bjoc.1999.0780

### Lack of MHC class I surface expression on neoplastic cells and poor activation of the secretory pathway of cytotoxic cells in oral squamous cell carcinomas

**I Cruz[1,2], CJLM Meijer[1], JMM Walboomers[1], PJF Snijders[1] and I Van der Waal[2]**

[1]Unit of Molecular Pathology, Department of Pathology; [2]Department of Oral & Maxillofacial Surgery/Oral Pathology/ACTA, University Hospital Vrije Universiteit, De Boelelaan 1117, 1081 HV Amsterdam, The Netherlands

**Summary** Cytotoxic T lymphocytes (CTLs) and natural killer (NK) cells use the secretory pathway of perforin/granzymes to kill their target cells. In contrast to NK cells, CTL responses are MHC class I restricted. In this study we analysed the relative activation of CTL and NK cells in relation with MHC class I expression on oral squamous cell carcinomas (OSCCs). MHC class I expression was investigated in 47 OSCCs by immunohistochemistry using HCA2, HC10 and β2-m antibodies. The presence of CTLs, NK cells, and its activation, was investigated in 21 of these OSCCs using respectively, CD8, CD57 and GrB7 antibodies. The Q-Prodit measuring system was used for quantification of cytotoxic cells. All OSCCs showed weak or absent staining of β2-m on the cell surface. The absence of β2-m was significantly associated with absent expression of MHC class I heavy chain as detected by HC10 antibody ($P = 0.004$). In tumour infiltrates CTLs always outnumbered NK cells, as reflected by the ratio CD57/CD8 being always inferior to one (mean: 0.19; SD: 0.15). The proportion of activated cytotoxic cells as detected by granzyme B expression was generally low (mean: 8.6%; SD 8.9). A clear correlation between MHC class I expression and the relative proportion of NK cells/CTLs was not found. This study shows that the majority of OSCCs show weak or absent expression of MHC class I molecules on the cell surface, possibly due to alterations in the normal β2-m pathway. The low proportion of granzyme B-positive CTLs/NK cells indicates that the secretory pathway of cytotoxicity is poor in these patients. The lack of correlation between MHC class I expression and CTL/NK cell activation as detected by granzyme B expression suggests that, next to poor antigen presentation, also local factors seem to determine the final outcome of the cytotoxic immune response. © 1999 Cancer Research Campaign

**Keywords:** oral carcinoma; MHC class I; β2-microglobulin; CTLs; NK cells; granzyme B

CD8+ cytotoxic T lymphocytes (CTLs) and natural killer (NK) cells play a major and complementary role in eliminating virally infected or otherwise antigenically modified cells (e.g. neoplastic cells) (Kos and Engleman, 1996). CTL responses are ‘MHC class I restricted’, as their activation is dependent on class I major histocompatibility molecules (MHC). MHC class I molecules transport peptides derived from proteins produced intracellularly to the surface of the cell, allowing recognition by specific T-cell receptors (TCRs) and CD8 molecules present on the surface of CTLs (Parham, 1995). MHC class I molecules consist of a polymorphic heavy chain, encoded within the classical MHC loci (HLA-A,B,C), which is stablized by a constant non-covalent associated light chain, β2-microglobulin (β2-m).

In contrast to CTLs, natural killer (NK) cells do not harbour TCRs on the cellular membrane, and their activation does not require MHC class I expression by the target cell, neither do they develop immunological memory (Yokoyama, 1995). Instead, and in addition to receptors that receive stimulatory signals (e.g. NKR-P1, CD2, CD16, CD69), NK cells have killer-cell inhibitory receptors (KIRs) that recognize polymorphic determinants on MHC class I molecules of their target cells. Some of the KIRs identified belong to the immunoglobulin superfamily and specifically recognize groups of HLA-C (e.g. p58), HLA-B (e.g. p70) and HLA-A alleles (p140) (Mingari et al, 1998). Other KIRs belong to the C-type lectin superfamily (e.g. CD94/NKG2A) and display a less well-defined allele specificity (Mingari et al, 1998). In contrast to CTLs which bear only one TCR per CTL, in NK cells several KIRs may be present per NK cell. Only when their target cells fail to express the relevant MHC class I allele(s), NK cells will be released from the inhibitory signals conveyed by the respective KIRs, and full activation of NK cells may ensue (Yokoyama, 1995; Lanier and Phillips, 1996).

When proper activation of cytotoxic cells occurs, killing of the target cell may occur. Two main pathways of fast-acting cytotoxicity have been described for CTLs, both activating the caspase cascade and resulting in apoptosis of the target cell (Berke, 1997). In the ‘secretory pathway’ perforin and granzymes present in granules of the cytotoxic cell, are released in the direct vicinity of the target cell. Perforin forms pores in the membrane of the target cell, allowing the entrance of granzyme B in its cytoplasm, which in turn will activate the caspase cascade (Froelich et al, 1998). In the ‘non-secretory pathway’, Fas ligand (Fas-L) expressed on the surface of the cytotoxic cell upon activation, will bind Fas when this is expressed on the surface of the target cell (Berke, 1997). This interaction will also result in the activation of the caspase cascade with subsequent apoptosis of the target cell. According to some reports NK cells only use the ‘secretory pathway’ as a mechanism of cytotoxicity (van den Broek et al, 1995; Kagi et al, 1996).

*Received 3 December 1998*
*Revised 21 April 1999*
*Accepted 13 May 1999*

*Correspondence to:* CJLM Meijer

In order to obtain more insight into the mechanisms that neoplastic cells use to evade the immune response, we analysed MHC class I expression in neoplastic cells of oral squamous cell carcinomas (OSCCs) using antibodies to the polymorphic heavy chains (HCA2 and HC10) and β2-m. Moreover, in relation with MHC class I expression we analysed the presence of CD8- and CD57-positive cells, and investigated whether the granzyme pathway was activated.

### MATERIALS AND METHODS

### Patients and tissues

Forty-seven formalin-fixed, paraffin-embedded surgical OSCC specimens from patients that were seen at the Department of Oral & Maxillofacial Surgery and Otorhinolaryngology from the Free University Hospital, Amsterdam, The Netherlands, were evaluated in this study. In 23 of these OSCCs, tumour adjacent normal or dysplastic mucosa was present in the same paraffin block of the carcinoma and was evaluated for MHC class I expression. Histologically normal epithelium at the surgical resection margin of six of these OSCCs and present in a different paraffin block from that containing the carcinoma, and eight clinically and histologically normal epithelium from healthy individuals subjected to third molar extractions, were also analysed for MHC class I expression.

### Immunohistochemical analysis

Four-micrometre sections from formalin-fixed, paraffin-embedded tissues were mounted on poly-L-lysine, or 3-amino-propyl-triethoxy-silane (APES)-coated slides. Consecutive sections were used as negative control of the immunohistochemical (IHC) reaction and for haematoxyline and eosin (H&E) staining to confirm the diagnosis.

The streptavidin–biotin complex immunoperoxidase technique used was previously described in detail (Cruz et al, 1998). Microwave antigen retrieval was performed for HCA2 in ‘target unmasking fluid’ (TUF) at 95°C, and for CD8, CD57 and GrB7 in citrate at 100°C (3 × 5 min). Primary antibodies were incubated, at room temperature, for 1 h: (a) monoclonal antibody (mAb) HCA2 (1:500) recognizing preferentially HLA-A locus products (Stam et al, 1990); (b) mAb HC10 (1:1000), recognizing preferentially HLA-B/C locus products (Stam et al, 1986); (c) polyclonal rabbit-anti-β2-microglobulin (Dako) (1/50), recognizing the constant/light chain of MHC class I molecule; (d) anti-CD8 (Clone C8144B, Dako) (1/50), a mouse monoclonal antibody which recognizes the CD8 molecule on the surface of cytotoxic/suppressor T-cells; (e) anti-CD57 (Zymed) (1/25), a mouse monoclonal which recognizes the CD57, an antigen associated with NK cells and a subset of human T-cells; (f) GrB7 (1/500), monoclonal antibody raised against recombinant granzyme B protein and specific for human granzyme B (Kummer et al, 1993, 1995). For β2-m, the only polyclonal antibody used, normal swine serum (1:10) and swine anti-rabbit (1:200) were used as normal serum and secondary antibody respectively. After the secondary antibody all cases were incubated with streptavidin–biotin complex horseradish peroxidase (1:200) for 1 h. The catalysed reporter deposition (CARD) method (Bobrow et al, 1989) was used for signal amplification of CD8 IHC. Negative controls consisting of phosphate-buffered saline (PBS) instead of primary antibody, and sections of a hyperplastic tonsil used as positive control, were included in each experience.

### Quantification of IHC results

MHC class I status of the lesions was scored under conventional light microscopy as follows. Cells were considered positive only when the staining was found on the cellular membrane. The MHC class I expression pattern of normal or dysplastic oral mucosa was classified in three categories: (–): no staining apparent; (+): staining confined to the basal/parabasal layers of the epithelium; (++): staining reaching spinous layer; (+++): staining reaching superficial layers of the epithelium.

The staining intensity of non-malignant oral epithelium adjacent to carcinoma, and the staining of inflammatory infiltrate, were used as a reference to define strong and weak staining in OSCCs. MHC class I expression patterns of individual carcinoma cases were divided into three categories, according to the intensity and number of positive cells: (–): less than 25% positive cells; (+/–): more than 25% and less than 75% positive cells, or more than 75% weakly positive cells; (+): more than 75% strong positive cells. For statistical analysis the categories (+/–) and (+), as defined above, were considered together defining a single category of ‘positive cases’.

To estimate the proportion of CTLs, NK cells and activated cytotoxic cells, CD8, CD57 and GrB7 positive cells were quantified using a commercially available interactive video overlay based measuring system (Q-Prodit, Leica, Cambridge, UK) (Brinkhuis et al, 1995). Neoplastic areas and respective inflammatory infiltrate were selected by us, under low power view. One hundred fields of vision (FOV) were evaluated using systematic random sampling, whereby the first FOV was chosen at random within the measurement area, and the subsequent fields were chosen systematically by adjusting the distance between the FOV in proportion to the area of tissue considering for sampling. These selections were made using an automated scanning stage controlled by Prodit. Eight points were counted per FOV using a Weibel test grid under a 40× objective (final magnification in the computer screen: 1200×). In each case, the numbers of CD8+, CD57+ and granzyme B+ cells were counted in similar areas of the carcinoma. The ratio between NK cells and CTLs was estimated by calculating the ratio between CD57+ cells and CD8+ cells in similar areas. The proportion of activated cytotoxic cells was estimated by calculating the ratio between granzyme B+ cells and the total number of CD57+ and CD8+ cells in similar areas. The proportion of activated CTLs was ‘(over)estimated’ by calculating the ratio between granzyme B+ cells and CD8+ cells in similar areas.

### Statistical analysis

Statistical analysis was performed using SPSS 7.0 software. Fisher’s exact test was used to compare cell surface expression of MHC class I heavy chains and β2-microglobulin. The ratio between NK cells and CTLs, and the proportion of activated cytotoxic cells as detected by granzyme B expression, were compared with cell surface expression of MHC class I using the Mann–Whitney two-sample test and the Wilcoxon signed rank test. Values were considered significantly different when $P$ was less than 0.05.

© 1999 Cancer Research Campaign

**Table 1** Clinicopathological data, MHC class I expression on tumour cells and proportion of (activated) cytotoxic cells

| Tn | Gend/Age | Locat | Grade | β2-m | HC10 | HCA2 | CD57:CD8 | GrB7/CD8+CD57 (%) | GrB7/CD8 (%) |
|---|---|---|---|---|---|---|---|---|---|
| 1 | F/63 | T | W-M | – | – | – | 0.09 | 0 | 0 |
| 2 | M/45 | OL | P | – | – | – | 0.07 | 0 | 0 |
| 3 | M/60 | OL | W-M | – | – | – | 0.08 | 37 | 46 |
| 4 | F/87 | FM | M-P | – | – | – | 0.11 | 3 | 3 |
| 5 | M/71 | T/FM | W-M | – | – | – | | | |
| 6 | F/55 | FM | W-M | – | – | – | | | |
| 7 | M/77 | OL | W | – | – | – | | | |
| 8 | M/70 | FM | M | – | – | +/– | 0.27 | 22 | 27 |
| 9 | M/72 | T | M | – | – | +/– | 0.13 | 15 | 16 |
| 10 | F/49 | FM | W-M | – | – | +/– | | | |
| 11 | F/75 | T | M | – | – | + | | | |
| 12 | M/67 | T | M | – | +/– | +/– | 0.15 | 7 | 9 |
| 13 | M/48 | OL | M | – | +/– | +/– | 0.22 | 10 | 13 |
| 14 | M/58 | OL | M-P | – | +/– | +/– | | | |
| 15 | M/69 | T | W-M | – | +/– | +/– | | | |
| 16 | F/51 | FM | W-M | – | +/– | +/– | | | |
| 17 | M/75 | T | W-M | – | +/– | nd | | | |
| 18 | M/59 | T | W-M | nd | +/– | – | 0.45 | 4 | 6 |
| 19 | M/81 | T | M | nd | – | – | 0.08 | 7 | 10 |
| 20 | M/76 | OL | W-M | nd | nd | – | | | |
| 21 | F/86 | T | W-M | +/– | nd | – | | | |
| 22 | F/69 | T | W-M | +/– | – | nd | | | |
| 23 | F/56 | OL | M | +/– | – | – | 0.29 | 11 | 13 |
| 24 | M/62 | T | M | +/– | – | +/– | 0.05 | 1 | 1 |
| 25 | M/59 | FM | M | +/– | – | +/– | | | |
| 26 | M/55 | T | W-M | +/– | – | +/– | | | |
| 27 | F/45 | OL | M-P | +/– | +/– | – | 0.10 | 6 | 8 |
| 28 | F/63 | T | W | +/– | +/– | – | 0.63 | 2 | 3 |
| 29 | F/80 | T | M | +/– | +/– | – | 0.33 | 17 | 24 |
| 30 | F/73 | OL | W-M | +/– | +/– | – | | | |
| 31 | M/52 | OL | W | +/– | +/– | – | | | |
| 32 | F/47 | OC | M | +/– | +/– | nd | | | |
| 33 | F/55 | T | W-M | +/– | +/– | +/– | 0.16 | 8 | 10 |
| 34 | F/81 | OL | W | +/– | +/– | +/– | 0.04 | 1 | 1 |
| 35 | F/82 | T | M | +/– | +/– | +/– | | | 27 |
| 36 | M/39 | T | W | +/– | +/– | +/– | | | |
| 37 | M/63 | FM | W | +/– | +/– | +/– | | | |
| 38 | M/70 | T | W-M | +/– | +/– | +/– | | | |
| 39 | M/81 | T | W-M | +/– | +/– | +/– | | | |
| 40 | M/68 | T | W-M | +/– | +/– | +/– | | | |
| 41 | F/57 | T | W | +/– | +/– | + | | | |
| 42 | M/59 | OL | W | +/– | + | +/– | 0.12 | 5 | 6 |
| 43 | F/79 | T | W | +/– | + | +/– | 0.34 | 8 | 11 |
| 44 | M/48 | T | W-M | +/– | + | +/– | | | |
| 45 | F/28 | T | W-M | +/– | + | + | | | |
| 46 | F/84 | FM | M | +/– | + | + | 0.12 | 7 | 8 |
| 47 | M/56 | T | W-M | +/– | + | + | | | |

Tn = Tumour number. Gend/Age = Gender/Age: M = Male; F = Female. Locat = Location of tumour: T = Tongue; FM = Floor of mouth; OL = Other location (within the oral cavity); OC=Oral cavity (location not specified). Grade = grade of differentiation: W=Well dif. SCC; W-M=Well to moderately dif. SCC; M=Moderately dif. SCC; M-P=Moderately to poor dif. SCC; P=Poor dif. SCC. β2-m, Hc10/HcA2 = MHC class I light and heavy chain expression at the surface of neoplastic cells: (–), (+/–), (+) = score system defined in Materials and Methods. CD57/CD8: ratio between number of NK cells and number of CTLs; GrB7/CD8+CD57: percentage of activated cytotoxic cells; GrB7/CD8: percentage of activated CTLs (overestimated).

### RESULTS

Table 1 shows the most relevant clinical and pathological data of the tumour cases. The MHC class I expression on the surface of neoplastic cells, and the ratio between NK cells and CTLs, percentage of activated cytotoxic cells and the percentage of activated CTLs are presented in Table 1.

### MHC class I expression

All tumours exhibited strongly MHC class I immunopositive inflammatory cells validating the success of the immune technique. Negative controls, consisting of PBS or IgG of the same subclass instead of the primary antibody, confirmed the specificity of the membranous staining.

*© 1999 Cancer Research Campaign* 

**Table 2** MHC class I expression, as detected by β2-m and HC10 antibodies, in non-malignant oral mucosa

| | β2-m | | | | HC10 | | | |
|---|---|---|---|---|---|---|---|---|
| | – | + | ++ | +++ | – | + | ++ | +++ |
| NOM | 0 | 1 | 7 | 0 | 2 | 6 | 0 | 0 |
| (Healthy indiv.) | | 12.5% | 87.5% | | 25% | 75% | | |
| NOM | 0 | 2 | 3 | 1 | 0 | 3 | 2 | 1 |
| (Res.Mar.OSCC) | | 33% | 50% | 17% | | 50% | 33% | 17% |
| NOM/DYS | 0 | 0 | 10 | 13 | 0 | 2 | 13 | 8 |
| (Adj.Muc.OSCC) | | | 43% | 57% | | 9% | 56% | 35% |

NOM (Healthy indiv.): clinically and histologically normal oral mucosa from healthy individuals; NOM (Res.Mar.OSCC): histologically normal mucosa from the surgical resection margin of OSCC; NOM/DYS (Adj.Muc.OSCC): histologically normal or dysplastic epithelium immediately adjacent to OSCC. (–): no staining apparent; (+) staining confined to basal/parabasal cell layers; (++): staining reaching spinous layer of the epithelium; (+++): staining reaching superficial layers of the epithelium.

### MHC class I expression in non-malignant mucosa

The MHC class I staining patterns of non-malignant mucosa obtained from healthy individuals and mucosa adjacent to and distant from the tumour in OSCC patients are depicted in Table 2. The β2-m staining was generally stronger than the staining obtained with HC10 in all three groups of non-malignant mucosa analysed (Table 2). In addition, increasing staining was observed with both β2-m and HC10 antibodies, when comparing normal mucosa of healthy individuals with normal mucosa in OSCCs' resection margins, and when comparing the latter with mucosa immediately adjacent to OSCCs (Table 2). However, a considerable variation in MHC class I expression occurred among individuals. Therefore, we also compared the staining patterns obtained in the OSCC's resection margin and in the mucosa adjacent to OSCC of the same patient, in six cases where this data was available. In four cases (67%) there was an increase in β2-m and HC10 staining in the tumour adjacent mucosa when compared to the more distant and histologically normal epithelium of the tumour resection border. In the remaining two cases (33%) a similar staining pattern was found in adjacent mucosa and the histologically normal resection margin of OSCC (++/++ and +++/+++ respectively).

Non-malignant mucosa immediately adjacent to carcinoma often showed strong expression of MHC class I molecules (Figure 1, A1, A2, C1, C2), either in dysplastic or histologically normal epithelium, and independently of the pattern of MHC class I expression in the adjacent carcinoma. Besides the basal epithelial layer, the upper spinous layers of the non-malignant epithelium were often stained, even with antibodies against MHC class I heavy chains (Figure 1, A,C). This was in contrast to the weak staining found in normal mucosa of healthy individuals that, when present, was confined to the basal/parabasal epithelial layers (Table 2).

We also compared MHC class I expression on the non-malignant mucosa adjacent to carcinoma with the staining observed in the respective OSCC of the same patient, in 23 cases where this data was available. Only in three out of these cases (13%), the strong expression detected in the adjacent non-malignant mucosa was maintained in the respective OSCC. These results were only obtained with the HC10 antibody (Figure 1 C2/D2). For β2-m the respective carcinoma showed weak staining on the tumour cells, in spite of the fact that the tumour adjacent mucosa showed strong staining (Figure 1 C1/D1). In the remaining twenty cases the tumour showed weak or absent expression of MHC class I, either by using HC10 or β2-m.

**Table 3** Association between light (β2-m) and heavy (HLA-B/C) chains of MHC class I, at the cell surface of OSCCs, as detected by immunohistochemistry

| | | β2-M (Light chain) | | | |
|---|---|---|---|---|---|
| Heavy chain | | (–) | (+) | ND | *P*-value* |
| HC10 (*n* = 43) | (–) | 11 (69%) | 5 (31%) | 1 | *P*=0.004 |
| | (+) | 6 (22%) | 21 (78%) | 1 | |
| | ND | – | 1 | 1 | |

HC10: monoclonal antibody that detects preferentially HLA-B/C heavy chains of MHC class I β2-m: polyclonal antibody that detects the light chain (β2-m) of MHC class I molecules. (–): <25% + cells; (+): >25% positive cells; ND: not done. *statistics performed using Fisher's exact test.

### *MHC class I expression in OSCCs*

All carcinomas analysed with β2-m antibody (*n* = 44) showed weak or absent β2-m staining at the surface of the neoplastic cells. Seventeen (39%) showed less than 25% positive tumour cells (score-, as previously defined) (Figure 1, B1), and 27 carcinomas (61%) showed more than 25% positive tumour cells, usually weakly stained (score +/–) (Figure 1, D1 and Table 1).

Out of 45 carcinomas analysed with HC10, 39 (87%) showed weak or absent MHC class I heavy chain expression in the cellular membrane. Seventeen (38%) were scored (–), and 22 (49%) were scored (+/–). Six carcinomas (13%) were strongly positive (score +) (Figure 1, D2 and Table 1). A significant association was found between immuno-negative/positive staining for β2-m and immuno-negative/positive staining for HC10 respectively ($P = 0.004$) (Table 3).

Out of 44 carcinomas analysed with HCA2, 39 (89%) showed weak or absent expression of MHC class I heavy chain in the cellular membrane. Seventeen (39%) were scored (–) (Figure 1, B2), and 22 (50%) were scored (+/–). Five carcinomas (11%) were scored (+) (Table 1). No significant association was found between HCA2 and β2-m immunostaining ($P = 0.332$).

### *(Co-)expression of MHC class I light and heavy chains, at the surface of neoplastic cells*

Forty carcinomas were analysed both with the antibody directed against MHC class I light chain (β2-m) and with the antibodies against MHC class I heavy chains (HC10 and HCA2). In the

 © 1999 Cancer Research Campaign

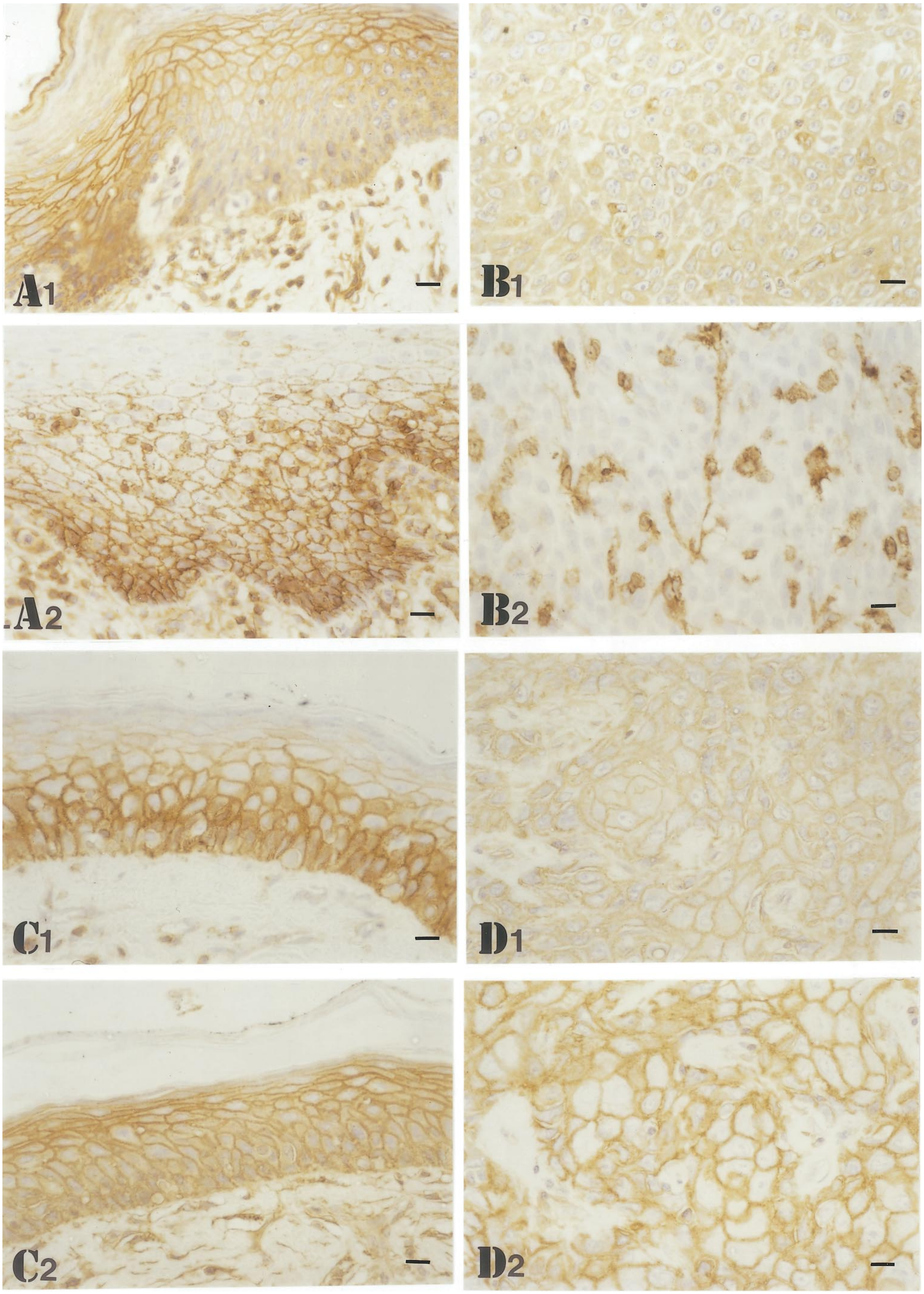

**Figure 1** (**A**) Non-malignant mucosa adjacent to OSCC (Table 1, T4) showing strong expression of MHC class I light and heavy chains in the cell membrane; (A1) IHC performed with β2-m antibody; (A2) IHC performed with HCA2 antibody. (**B**) OSCC adjacent to the non-malignant mucosa shown in A (Table 1, T4) showing neoplastic cells without any membranous staining (–) for MHC class I light and heavy chains: (B1) IHC performed with β2-m antibody shows no membranous staining, although cytoplasmatic staining is present; (B2) IHC performed with HCA2 antibody shows neither membranous or cytoplasmatic staining in the tumour cells. Dendritic cells are positive. (**C**) Non-malignant mucosa adjacent to OSCC (Table 1, T46) showing strong expression of MHC class I light and heavy chains in the cell membrane; (C1) IHC performed with β2-m antibody; (C2) IHC performed with HC10 antibody. (**D**) OSCC adjacent to the non-malignant mucosa shown in C (Table 1, T46) showing: (D1) weak expression (+/–) of the MHC class I light chain (β2-m) in the cell membrane; (D2) strong membranous staining (+) for the MHC class I heavy chain, as detected by HC10 antibody. Size bars represent 30 μm

*© 1999 Cancer Research Campaign* 

remaining seven cases, results for β2-m ($n$ = 3), HC10 ($n$ = 2) and HCA2 ($n$ = 3) were not available, and these cases were excluded from this analysis. In seven carcinomas (17.5%) a simultaneous loss of light and heavy chains was apparent, as reflected by absence of staining with β2-m and HC10/HCA2 antibodies respectively (Table 1, cases T1–T7). In nine cases (22.5%) the heavy chain could be detected on the cell surface, in the absence of the light chain: these included four cases in which only HCA2 gave positive results (Table 1, cases T8–T11) and five cases that were positive with both HC10 and HCA2 antibodies (Table 1, cases T12–T16). In one case (2.5%) the light chain could be detected in the absence of detectable heavy chains (Table 1, case T23). In eight cases (20%) expressing the light chain at the cell surface only heavy chains corresponding to a certain HLA loci (HLA-B/C versus HLA-A, as reflected by HC10 or HCA2 staining respectively) could be detected: three cases were detected with HCA2 (Table 1, cases T24–T26) and five cases were detected with HC10 (Table 1, cases T27–T31). Finally, in 15 cases (37.5%) both light and heavy chains of both HLA loci (HLA-B/C and HLA-A) were detected in the surface of neoplastic cells (Table 1, cases T33–T47).

### Proportion of activated cytotoxic cells (CTL and NK cells) infiltrating OSCCs as detected by granzyme B expression

In order to estimate the proportion of activated cytotoxic cells (CTLs and NK cells) the expression of CD8, CD57 and granzyme B7 was analysed, by IHC, in consecutive sections of 21 OSCCs tested for MHC class I expression. The results are depicted in Table 1 and a representation of them is shown in Figure 2.

The ratio between NK cells and CTLs infiltrating the tumour, was estimated by calculating the ratio between CD57+ and CD8+ cells in consecutive sections of the OSCCs. CTLs always outnumbered NK cells, although the relative proportion of these two varied among different OSCCs. The ratio between NK cells and CTLs ranged from 0.04 to 0.63 (mean: 0.19; SD 0.15) (Table 1 and Figure 2 A2,A3,B2,B3).

The proportion of activated cytotoxic cells (CTLs and NK cells) was estimated by calculating the proportion of CD8+ and CD57+ cells that were granzyme B-positive. This proportion indicated that 0% to 37% of the cytotoxic cells were activated in the OSCCs analysed (mean: 8.6%; SD 8.9) (Table 1 and Figure 2 A4, B4). In addition, and since the CD8+ population was the population of cytotoxic cells more represented in the OSCC, we also calculated the proportion of CD8+ cells that were granzyme B+, bearing in mind that this ratio may overestimate the proportion of activated CTLs due to inclusion of some activated cells that belong to the NK cell population, particularly in MHC class I-negative cases. These proportion ranged from 0% to 46% (mean: 11.52%; SD 11.35) (Table 1).

No correlation between MHC class I expression and the relative proportion of NK cells/CTLs was found (Table 1 and Figure 2). Similarly, no correlation was found between the proportion of activated cytotoxic cells, as detected by granzyme B/CD57+CD8, and the relative expression of MHC class I (Table 1). Nevertheless, in general, the proportion of activated cytotoxic cells was poor, as reflected by the mean value for granzyme B+ cytotoxic cells (8.6%). This is in agreement with the finding that in general all OSCCs analysed had alterations in the MHC class I expression.

### DISCUSSION

In the present study we found weak or absent expression of MHC class I molecules on the surface of neoplastic cells, in the majority of OSCCs studied, probably due to alterations in the β2-m pathway. In addition, activation of cytotoxic cells (i.e. CTLs and NK cells) as detected by granzyme B expression in CD8+ and CD57+ cells, was remarkably low.

### MHC class I expression in non-malignant mucosa

Inflammatory infiltrating cells were always strongly positive and were used as an internal positive control for each sample analysed. The MHC class I expression pattern of normal oral mucosa from healthy individuals was compared with normal oral mucosa present in the resection margin of OSCCs and with oral mucosa immediately adjacent to OSCCs. Although inter-individual variation occurred, generally, the highest MHC class I expression was found immediately adjacent to OSCCs, either in normal or dysplastic epithelium, and the lowest expression was found in normal oral mucosa from healthy individuals. Intermediate levels of MHC class I expression occurred in the histologically normal oral mucosa present in the resection margins of OSCCs. This suggests up-regulation of MHC class I expression during the initial steps of oral carcinogenesis, although the extent to which this occurs vary among different individuals. The generalized weak expression of MHC class I found among OSCCs, suggests that this mechanism of up-regulation fails upon progression towards malignancy. We tried to illustrate this hypothetical mechanism in Figure 3. Interestingly we found a different expression of MHC class I molecules as detected by β2-m and HC10 staining in non-malignant epithelium and carcinoma (Figure 3). In the former, the β2-m staining was invariably stronger than the HC10 staining, whereas in carcinoma the HC10 staining was similar to or stronger than the β2-m staining (Figure 3). This indicates that the differences in staining of light and heavy chains of MHC class I are not a consequence of differences in the sensitivities of the respective antibodies, but rather reflect differential activation, or deregulation of the respective genes or gene products during oral carcinogenesis.

### MHC class I expression in OSCCs

All OSCCs showed decreased expression of β2-m on the surface of the neoplastic cells, taking as reference the strong intensity of staining in the non-malignant mucosa adjacent to carcinomas. These findings are in agreement with those of Prime et al (1987) who, using a semi-quantitative technique to measure MHC class I expression in OSCC, reported a significant loss of β2-m on the surface of OSCC. A significant association was found between β2-m staining and HC10 staining, with loss of β2-m being associated with loss of heavy chain as detected by HC10 antibody. However, this association was not found when the β2-m results were compared with HCA2 results. Our explanation for this finding could be that heavy chains recognized by HCA2 antibody (mainly of the HLA-A type) might be more stable at the cell surface in the absence of β2-m than the heavy chains recognized by HC10 (mainly of the HLA-B/C types). The underlying mechanism of β2-m weak expression could involve deletion or mutation in the β2-m gene, in analogy with other well studied tumours (Klein et al, 1967; Rosa et al, 1983; D'Urso et al, 1991; Bicknell et al, 1994). Alternatively, it could result from a post-translational

 © 1999 Cancer Research Campaign

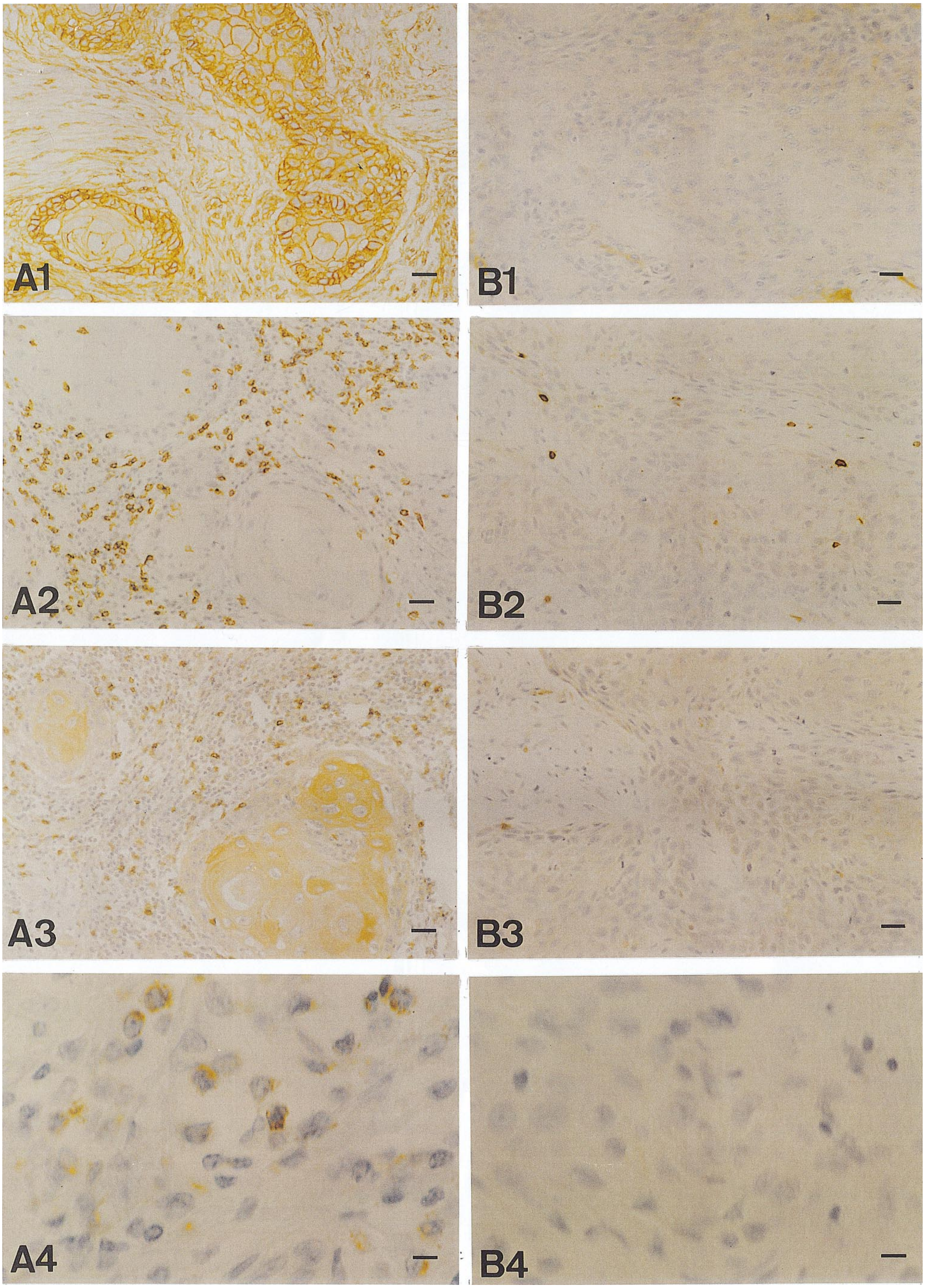

**Figure 2** (**A**) Well-differentiated OSCC (Table 1, T43) showing: (A1) strong expression of MHC class I heavy chain as detected by HC10 antibody; (A2) numerous CD8+ lymphocytes surrounding tumour islands; (A3) less numerous amount of CD57+ cells surrounding tumour nests; (A4) granzyme B + lymphocytes infiltrating neoplastic areas. (**B**) Well to moderately-differentiated OSCC (Table 1, T1) showing: (B1) no expression of MHC class I heavy chain in the cell membrane, as detected by HC10 antibody; (B2) scarce CD8+ cells infiltrating tumour areas; (B3) scarcely isolated CD57+ cells in the tumour area; (B4) absence of granzyme B + cells within areas of tumour infiltrating lymphocytes. Size bars represent 60 μm (A1–A3, B1–B3) and 15 μm (A4, B4)

*© 1999 Cancer Research Campaign*

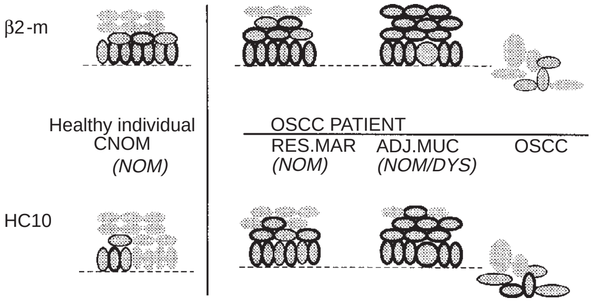

**Figure 3** Schematic representation of MHC class I expression patterns in normal oral mucosa of healthy individuals and in epithelium of tumour resection margins, tumour adjacent mucosa and OSCC. CNOM: clinically normal oral mucosa of non-cancer subject; NOM: histologically normal mucosa; RES.MAR: surgical resection margin of carcinoma; ADJ.MUC: tumour adjacent normal (NOM) and dysplastic (DYS) mucosa; OSCC: oral squamous cell carcinoma. The dotted line (----) represents the basal lamina mechanism such as sequestering of the β2-m protein by a viral or other protein, in analogy with the mechanism described for cytomegalovirus down-regulation of MHC class I molecules (Chapman and Bjorkman, 1998).

In some OSCCs MHC class I heavy chain was detected in the cell surface, in spite that no β2-m could be detected. We anticipated that these OSCCs would not be efficient in activating CTL responses, due to conformational alterations and/or impaired stability of the complexes. Therefore, we analysed granzyme B expression, as a marker of activation of cytotoxic cells. We choose this activation marker of cytoxicity, because the secretory pathway of perforin/granzymes is assumed to play a key role in the cytotoxic immune response against tumour cells.

### Cytotoxic activation of CTLs and NK cells in OSCCs

Since CTL responses are MHC class I restricted, we already expected poor CTL activation, after demonstration of MHC class I down-regulation in OSCCs. Interestingly, we also found a considerably low proportion of NK cells infiltrating these tumours. These numbers may even be lower than estimated, since CD57 monoclonal antibody can detect a subset of human T lymphocytes in addition to NK cells.

Although we found poor activation of CTLs, as detected by granzyme B expression, a correlation between the number of activated CTLs and MHC class I expression was not found when comparing individual cases. As pointed out before, peptide presentation by MHC class I molecules, although being essential, is not the only factor that plays a role in the activation of CTLs. Similarly, it is expected that the absence of MHC class I molecules will not be the exclusive factor to determine NK cell activation. The presence or absence of co-stimulatory molecules (e.g. B7) in the antigen-presenting cell, as well as the type of ligands on CTLs with which it interacts (e.g. CD28 versus CTLA-4), may result in enhancement or inhibition of the cytotoxic cell to kill its target (Chambers and Allison, 1997). Moreover, the type of cytokines produced by CD4+ helper T-cells influence the type of immune response. In this context, the interaction of interleukin-2 (IL-2) with its receptor on the cytotoxic cell seems crucial for expansion of cytotoxic cells (Whiteside et al, 1996; Gomez et al, 1998), whereas IL-10 inhibits cellular immune responses (Ding et al, 1993). In addition, in a proportion of OSCCs, functional impairment of T lymphocytes infiltrating these tumours was found to be due to abnormalities in the zeta-chain of these lymphocytes (Reichert et al, 1998). Another mechanism of immune escape might occur when tumour cells (abnormally) express Fas-L acquiring the ability to induce apoptosis in Fas-expressing TILs. This has been shown to occur in oesophageal carcinoma (Bennet et al, 1998).

At present we do not know which of these mechanisms might explain the lack of a direct relationship between MHC class I expression and activation of cytotoxic cells (CTLs/NK cells).

The study of Bontkes et al (1997) gives some support to our findings. These authors analysed the proportion of activated CTLs and NK cells in cervical cancer and its prestages, by means of granzyme B staining, but they also did not find a correlation between granzyme B-expressing cells and the MHC class I expression in the tumours. Interestingly, in that study granzyme B + cells were always found in every tumour and the mean values of granzyme B-expressing cells was generally higher than the values found in this study. Indeed, in the cervical SCCs studied, the percentage of CD8+ lymphocytes that were positive for granzyme B varied between 15% and 100%. Since in both studies the antibody used to detect granzyme B was the same, these findings support studies indicating that the cellular immune response in patients with head and neck cancer is particularly poor (Wanebo et al, 1975; Lichtenstein et al, 1980).

In conclusion, this study shows that weak or absent expression of β2-m light chain of MHC class I molecules are extremely frequent in OSCCs, and likely to be an important mechanism of immune evasion for OSCC cells. This hypothesis is supported by the finding of very low numbers of activated CTLs and NK cells as detected by granzyme B expression. The fact that a clear correlation between the relative proportion of CTLs/NK cells and MHC class I expression was not always apparent in the individual cases suggest that local immune suppression conveyed by cytokines such as IL-10 (Ding et al, 1993), and other inhibitory local factors or functional abnormalities of TILs, are likely to play a role in immune evasion of the neoplastic cells in OSCC.

### ACKNOWLEDGEMENTS

The authors wish to thank Drs Jacqueline van der Wal and Kees Pieter Schepman for helpful collaboration in collecting clinical material and patient data, Thea Tadema for technical advice and laboratory facilities, Jane Brugghe for technical advice in the use of Q-Prodit, Dr Paul van Diest for reviewing statistical analysis and Prof. Peter Stern for helpful discussions. This work (I Cruz) was supported by the Portuguese National Agency for Research and Technology (JNICT) (PRAXIS XXI Programme).

### REFERENCES

Bennett MW, O'Connell J, O'Sullivan GC, Brady C, Roche D, Collins JK and Shanahan F (1998) The Fas counterattack in vivo: apoptotic depletion of tumour-infiltrating lymphocytes associated with Fas ligand expression by human esophageal carcinoma. *J Immunol* **160**: 5669–5675

Berke G (1997) Killing mechanisms of cytotoxic lymphocytes. *Curr Opin Hematol* **4**: 32–40

Bicknell DC, Rowan A and Bodmer WF (1994) β2-microglobulin mutations: a study of established colorectal cell lines and fresh tumours. *Proc Natl Acad Sci USA* **91**: 4751–4755

Bobrow MN, Harris TD, Shaughnessy KJ and Litt GJ (1989) Catalysed reporter deposition, a novel method of signal amplification. *J Immunol Methods* **125**: 279–285

*© 1999 Cancer Research Campaign*

Bontkes HJ, De Gruijl TD, Walboomers JMM, Van den Muysenberg AJC, Gunther AW, Scheper RJ, Meijer CJLM and Kummer JA (1997) Assessment of cytotoxic T lymphocyte phenotype using the specific markers granzyme B and TIA-1 in cervical neoplastic lesions. *Br J Cancer* **76**: 1353–1360

Brinkhuis M, Mogensen O, Bichel P and Baak JPA (1995) The significance of differences in prognostic value of quantitative pathologic features in FIGO stage III and IV serous adenocarcinoma of the ovary between a group of Danish patients and other groups. *Int J Gynecol Cancer* **5**: 355–360

Chambers CA and Allison JP (1997) Co-stimulation in T cell responses. *Curr Opin Immunol* **9**: 396–404

Chapman TL and Bjorkman PJ (1998) Characterization of a murine cytomegalovirus class I major histocompatibility complex (MHC) homolog: comparison to MHC molecules and to the human cytomegalovirus MHC homolog. *J Virol* **72**: 460–466

Cruz IB, Snijders PJF, Meijer CJ, Braakhuis BJ, Snow GB, Walboomers JM and Van der Waal I (1998) p53 expression above the basal cell layer in oral mucosa is an early event of malignant transformation and has predictive value for developing oral squamous cell carcinoma. *J Pathol* **184**: 360–368

Ding L, Linsley PS, Huang LY, Germain RN and Shevach EM (1993) IL-10 inhibits macrophage co-stimulatory activity by selectively inhibiting the up-regulation of B7 expression. *J Immunol* **151**: 1224–1234

D'Urso CM, Wang Z, Cao Y, Tatake R, Zeff RA and Ferrone S (1991) Lack of HLA class I antigen expression by cultured melanoma cells FO-1 due to a defect in β2-m gene expression. *J Clin Invest* **87**: 284–292

Froelich CJ, Dixit VM and Yang X (1998) Lymphocyte granule-mediated apoptosis: matters of viral mimicry and deadly proteases. *Immunol Today* **19**: 30–37

Gomez J, Gonzalez A, Martinez-A C and Rebollo A (1998) IL-2-induced cellular events. *Crit Rev Immunol* **18**: 185–220

Kagi D, Ledermann B, Burki K, Zinkernagel RM and Hengartner H (1996) Molecular mechanisms of lymphocyte-mediated cytotoxicity and their role in immunological protection and pathogenesis in vivo. *Annu Rev Immunol* **14**: 207–232

Klein E, Klein G, Nadkarni JS, Nadkarni JJ, Wigzell H and Clifford P (1967) Surface IgM specificity on cells derived from a Burkitt's lymphoma. *Lancet* **ii**: 1068–1070

Kos FJ and Engleman EG (1996) Immune regulation: a critical link between NK cells and CTLs. *Immunol Today* **17**: 174–176

Kummer JA, Kamp AM, Van Katwijn M, Brakenhof JP, Radosevic K, Van Leeuwen AM, Borst J, Verwey CL and Hack CE (1993) Production and characterization of monoclonal antibodies raised against recombinant human granzymes A and B showing cross reactions with the natural proteins. *J Immunol Methods* **163**: 77–83

Kummer JA, Kamp AM, Tadema TM, Vos W, Meijer CJLM and Hack CE (1995) Localization and identification of granzymes A and B-expressing cells in normal human lymphoid tissue and peripheral blood. *Clin Exp Immunol* **100**: 164–172

Lanier LL and Phillips JH (1996) Inhibitory MHC class I receptors on NK cells and T cells. *Immunol Today* **17**: 86–91

Lichtenstein A, Zighelboim J, Dorey F, Brossman S and Fahey JL (1980) Comparison of immune derangements in patients with different malignancies. *Cancer* **45**: 2090–2095

Mingari MC, Moretta A and Moretta L (1998) Regulation of KIR expression in human T cells: a safety mechanism that may impair protective T-cell responses. *Immunoly Today* **19**: 153–157

Parham P (1995) Antigen presentation by class I major histocompatibility complex molecules: a context for thinking about HLA-G. *Am J Reprod Immunol* **34**: 10–19

Prime SS, Pitigala-Arachchi A, Crane IJ, Rosser TJ and Scully C (1987) The expression of cell surface MHC class I heavy and light chain molecules in pre-malignant and malignant lesions of the oral mucosa. *Histopathology* **11**: 81–91

Reichert TE, Day R, Wagner EM and Whiteside TL (1998) Absent or low expression of the zeta chain in T cells at the tumour site correlates with poor survival in patients with oral carcinoma. *Cancer Res* **58**: 5344–5347

Rosa F, Berissi H, Weissenbach J, Maroteaux L, Fellous M and Revel M (1983) The β2-microglobulin RNA in human Daudi cell has a mutated initiation codon but is still inducible by interferon. *EMBO J* **2**: 239–243

Stam NJ, Spits H and Ploegh HL (1986) Monoclonal antibodies raised against denaturated HLA-B locus heavy chains permit biochemical characterization of certain HLA-C locus products. *J Immunol* **137**: 2299–2306

Stam NJ, Vroom ThM, Peters PJ, Pastoors EB and Ploegh HL (1990) HLA-A- and HLA-B-specific monoclonal antibodies reactive with free heavy chains in Western blots, in formalin-fixed, paraffin-embedded tissue sections and in cryo-immuno-electron microscopy. *Int Immunol* **2**: 113–125

Van den Broek MF, Kagi D, Zinkernagel RM and Hengartner H (1995) Perforin dependence of natural killer cell-mediated tumour control in vivo. *Eur J Immunol* **25**: 3514–3516

Wanebo HJ, Jun MY, Strong EW and Oettgen H (1975) T-cell deficiency in patients with squamous cell cancer of the head and neck. *Am J Surg* **130**: 445–451

Whiteside TL, Chikamatsu K, Nagashima S and Okada K (1996) Anti-tumor effects of cytotoxic T lymphocytes (CTL) and natural killer (NK) cells in head and neck cancer. *Anticancer Res* **16**: 2357–2364

Yokoyama WM (1995) Natural killer cell receptors. *Curr Opin Immunol* **7**: 110–120

*© 1999 Cancer Research Campaign*

## References

[PDF_00585] Bennett M. W., O'Connell J., O'Sullivan G. C., Brady C., Roche D., Collins J. K., Shanahan F. (1998). The Fas counterattack in vivo: apoptotic depletion of tumor-infiltrating lymphocytes associated with Fas ligand expression by human esophageal carcinoma.. J Immunol.

[PDF_00589] Berke G. (1997). Killing mechanisms of cytotoxic lymphocytes.. Curr Opin Hematol.

[PDF_00591] Bicknell D. C., Rowan A., Bodmer W. F. (1994). Beta 2-microglobulin gene mutations: a study of established colorectal cell lines and fresh tumors.. Proc Natl Acad Sci U S A.

[PDF_00594] Bobrow M. N., Harris T. D., Shaughnessy K. J., Litt G. J. (1989). Catalyzed reporter deposition, a novel method of signal amplification. Application to immunoassays.. J Immunol Methods.

[PDF_00617] Bontkes H. J., de Gruijl T. D., Walboomers J. M., van den Muysenberg A. J., Gunther A. W., Scheper R. J., Meijer C. J., Kummer J. A. (1997). Assessment of cytotoxic T-lymphocyte phenotype using the specific markers granzyme B and TIA-1 in cervical neoplastic lesions.. Br J Cancer.

[PDF_00621] Brinkhuis M., Mogensen O., Bichel P., Baak J.P.A. (1995). The significance of differences in prognostic value of quantitative pathologic features in FIGO stage III and IV serous adenocarcinoma of the ovary between a group of Danish patients and other groups.. Int J Gynecol Cancer.

[PDF_00625] Chambers C. A., Allison J. P. (1997). Co-stimulation in T cell responses.. Curr Opin Immunol.

[PDF_00627] Chapman T. L., Bjorkman P. J. (1998). Characterization of a murine cytomegalovirus class I major histocompatibility complex (MHC) homolog: comparison to MHC molecules and to the human cytomegalovirus MHC homolog.. J Virol.

[PDF_00631] Cruz I. B., Snijders P. J., Meijer C. J., Braakhuis B. J., Snow G. B., Walboomers J. M., van der Waal I. (1998). p53 expression above the basal cell layer in oral mucosa is an early event of malignant transformation and has predictive value for developing oral squamous cell carcinoma.. J Pathol.

[PDF_00638] D'Urso C. M., Wang Z. G., Cao Y., Tatake R., Zeff R. A., Ferrone S. (1991). Lack of HLA class I antigen expression by cultured melanoma cells FO-1 due to a defect in B2m gene expression.. J Clin Invest.

[PDF_00635] Ding L., Linsley P. S., Huang L. Y., Germain R. N., Shevach E. M. (1993). IL-10 inhibits macrophage costimulatory activity by selectively inhibiting the up-regulation of B7 expression.. J Immunol.

[PDF_00641] Froelich C. J., Dixit V. M., Yang X. (1998). Lymphocyte granule-mediated apoptosis: matters of viral mimicry and deadly proteases.. Immunol Today.

[PDF_00643] Gómez J., González A., Martínez-A C., Rebollo A. (1998). IL-2-induced cellular events.. Crit Rev Immunol.

[PDF_00649] Klein E., Klein G., Nadkarni J. S., Nadkarni J. J., Wigzell H., Clifford P. (1967). Surgace IgM specificity on cells derived from a Burkitt's lymphoma.. Lancet.

[PDF_00652] Kos F. J., Engleman E. G. (1996). Immune regulation: a critical link between NK cells and CTLs.. Immunol Today.

[PDF_00659] Kummer J. A., Kamp A. M., Tadema T. M., Vos W., Meijer C. J., Hack C. E. (1995). Localization and identification of granzymes A and B-expressing cells in normal human lymphoid tissue and peripheral blood.. Clin Exp Immunol.

[PDF_00654] Kummer J. A., Kamp A. M., van Katwijk M., Brakenhoff J. P., Radosević K., van Leeuwen A. M., Borst J., Verweij C. L., Hack C. E. (1993). Production and characterization of monoclonal antibodies raised against recombinant human granzymes A and B and showing cross reactions with the natural proteins.. J Immunol Methods.

[PDF_00645] Kägi D., Ledermann B., Bürki K., Zinkernagel R. M., Hengartner H. (1996). Molecular mechanisms of lymphocyte-mediated cytotoxicity and their role in immunological protection and pathogenesis in vivo.. Annu Rev Immunol.

[PDF_00663] Lanier L. L., Phillips J. H. (1996). Inhibitory MHC class I receptors on NK cells and T cells.. Immunol Today.

[PDF_00665] Lichtenstein A., Zighelboim J., Dorey F., Brossman S., Fahey J. L. (1980). Comparison of immune derangements in patients with different malignancies.. Cancer.

[PDF_00668] Mingari M. C., Moretta A., Moretta L. (1998). Regulation of KIR expression in human T cells: a safety mechanism that may impair protective T-cell responses.. Immunol Today.

[PDF_00671] Parham P. (1995). Antigen presentation by class I major histocompatibility complex molecules: a context for thinking about HLA-G.. Am J Reprod Immunol.

[PDF_00674] Prime S. S., Pitigala-Arachchi A., Crane I. J., Rosser T. J., Scully C. (1987). The expression of cell surface MHC class I heavy and light chain molecules in pre-malignant and malignant lesions of the oral mucosa.. Histopathology.

[PDF_00677] Reichert T. E., Day R., Wagner E. M., Whiteside T. L. (1998). Absent or low expression of the zeta chain in T cells at the tumor site correlates with poor survival in patients with oral carcinoma.. Cancer Res.

[PDF_00680] Rosa F., Berissi H., Weissenbach J., Maroteaux L., Fellous M., Revel M. (1983). The beta2-microglobulin mRNA in human Daudi cells has a mutated initiation codon but is still inducible by interferon.. EMBO J.

[PDF_00683] Stam N. J., Spits H., Ploegh H. L. (1986). Monoclonal antibodies raised against denatured HLA-B locus heavy chains permit biochemical characterization of certain HLA-C locus products.. J Immunol.

[PDF_00686] Stam N. J., Vroom T. M., Peters P. J., Pastoors E. B., Ploegh H. L. (1990). HLA-A- and HLA-B-specific monoclonal antibodies reactive with free heavy chains in western blots, in formalin-fixed, paraffin-embedded tissue sections and in cryo-immuno-electron microscopy.. Int Immunol.

[PDF_00693] Wanebo H. J., Jun M. Y., Strong E. W., Oettgen H. (1975). T-cell deficiency in patients with squamous cell cancer of the head and neck.. Am J Surg.

[PDF_00695] Whiteside T. L., Chikamatsu K., Nagashima S., Okada K. (1996). Antitumor effects of cytolytic T lymphocytes (CTL) and natural killer (NK) cells in head and neck cancer.. Anticancer Res.

[PDF_00698] Yokoyama W. M. (1995). Natural killer cell receptors.. Curr Opin Immunol.

[PDF_00690] van den Broek M. F., Kägi D., Zinkernagel R. M., Hengartner H. (1995). Perforin dependence of natural killer cell-mediated tumor control in vivo.. Eur J Immunol.

